# Anti-inflammatory reliever therapy for asthma using inhaled budesonide/formoterol as-needed with or without maintenance in South African children (AIR-SA 001): a description of a randomised clinical trial protocol

**DOI:** 10.1136/bmjresp-2025-003378

**Published:** 2025-11-17

**Authors:** ST Hlophe, NN Ndimande, N Ngobese, E Mkwanazi, K Bird, J Mbonigaba, K Otwombe, L Lebina, K Mortimer, R Masekela

**Affiliations:** 1 Department of Paediatrics and Child Health, College of Health Sciences University of KwaZulu Natal, Durban, South Africa; 2 Africa Health Research Instutute, Somkhele, Kwazulu Natal, South Africa; 3 University of KwaZulu-Natal, Durban, South Africa; 4 Faculty of Health Sciences, University of Witwatersrand, Johannesburg, South Africa; 5 Respiratory Medicine, Liverpool University Hospitals NHS Foundation Trust, Liverpool, UK

**Keywords:** Asthma, Paediatric asthma

## Abstract

**Background:**

Asthma is the most common non-communicable disease among children, with increasing prevalence. The current standard of care in high-income countries in adults and adolescents includes the use of combination inhaled corticosteroids (ICSs) with rapid-onset long-acting ß_2_ agonists (LABA) for all severities of asthma. The primary objective of this trial is to assess the efficacy of a budesonide/formoterol inhaler used ‘both as required, and regularly’ to reduce asthma exacerbations compared with the standard of care for asthma in children and adolescents.

**Methods:**

Children and adolescents aged 6–18 years with a diagnosis of asthma with at least one asthma exacerbation in the previous 12 months will be randomised to receive either budesonide/formoterol inhaler or the standard of care, which includes ICS and short-acting ß_2_ agonist (SABA). The primary outcome will be the number of severe asthma exacerbations over 1-year follow-up period. Secondary objectives will include evaluating the quality of life, lung function and health economic outcomes.

**Discussion:**

The current standard of care in South Africa recommends use of separate ICSs and SABA inhalers for asthma management in children with no recommendation for ICS/LABA in children under the age of 12 years for non-severe asthma. Budesonide/formoterol has transformed asthma treatment in high-income countries for use ‘as needed’ as anti-inflammatory reliever and for maintenance and reliever in adolescence, 12−18 years and adults. This strategy has been shown to reduce asthma exacerbations and hospitalisations. This trial will bridge the gap for the efficacy of budesonide/formoterol in children <12 years of age and address the economic arguments and safety of this approach for implementation in the lower to middle income countries. If this trial demonstrates positive results in the study population, it could provide strong scientific evidence and policy relevance to be adopted by policymakers for clinical implementation.

**Trial registration number:**

This study has been registered and approved by the South African Health Regulatory Authority 20231016, on 14 December 2023, KwaZulu Natal Health Research Committee KZ_202304_008 on 11 January 2024, University of KwaZulu Natal Biomedical Research Ethics Committee BREC/0000/5663/2023 on 6 February 2024, South African Clinical Trials Register DOH-27-032024-4778 on 14 March 2024, ClinicalTrial.gov NCT06429475 on 20 May 2024 and Pan African Clinical Trial Registry on 27 February 2025; the unique identification number for the registry is PACTR202502547023775.

**WHAT IS ALREADY KNOWN ON THIS TOPIC**

- Asthma is the most common non-communicable disease in children and asthma morbidity is high in Africa.

**WHAT THIS STUDY ADDS**

- In low to middle income countries (LMICs), the use of budesonide–formoterol is limited due to upfront high cost of these inhalers and limited evidence in younger population. Cost-effectiveness data are therefore lacking and necessary to inform the affordability of this approach in LMICs.

**HOW MIGHT THIS STUDY AFFECT RESEARCH, PRACTICE OR POLICY**

- If budesonide–formoterol demonstrates efficacy and cost effectiveness compared with standard of care in children, this will inform national and global practice of this approach in younger children.

## Introduction

### Background and rationale

 Asthma is a cause of substantial disability and death worldwide and it requires global attention and commitment to lessen its burden.^1^ A recent Global Asthma Network Phase I study reported a lifetime prevalence and current asthma rates at 34.5% and 21.3%, respectively.^2^ South Africa has the highest asthma prevalence in Africa, with one (21%) in five adolescents exhibiting asthma symptoms.

Mild-to-moderate asthma in children carries a disease burden globally, and until the uncertainties around management are addressed, this burden will remain.^3^ Previously, short-acting ß_2_ agonist (SABA) was used for mild intermittent asthma but there has been a shift driven by predominantly adult studies showing that SABA-only therapy is associated with an increased risk of asthma exacerbations and asthma mortality.^4^ A large body of randomised controlled clinical trial evidence from Symbicort Given as needed in Mild Asthma (SYGMA),^5 6^ Novel START^7^ and PRACTICAL^8^ has shown that use of budesonide/formoterol as needed (for exacerbations) for long-term controller treatment, compared with separate inhaled corticosteroid (ICS) and short-acting bronchodilators, reduces the number of asthma exacerbations and improves quality of life (QoL).

Budesonide/formoterol has transformed asthma treatment for use ‘as needed’ and for maintenance, as it improves symptom control and reduces exacerbations.^69^,^14^ Reasons for poor control are postulated to be due to limited availability and affordability of asthma inhalers and a lack of diagnosis, poorly coordinated asthma management, a lack of asthma knowledge in children, teachers and parents and stigma associated with this long-term condition.^1^

Essential medicines for treating asthma are largely unavailable and unaffordable in low to middle income countries (LMICs), and this is particularly true for ICS.^15^ Socioeconomic and environmental conditions, which influence behaviours and health, may be context specific. As such, the evidence from other regions may not be directly applicable to the South African context. There is a fundamental lack of clinical trial evidence to inform clinical care or policy for people with asthma in sub-Saharan Africa and South Africa.^16^ We are looking forward to the findings of the two randomised controlled trials done to determine the safety and efficacy of as-needed budesonide–formoterol in children with asthma.^17 18^

The proposed trial will provide important new clinical trial evidence about the clinical effectiveness and cost-effectiveness of a simple, pragmatic and intuitive approach with budesonide/formoterol compared with the standard of care in South Africa, in children 6–18 years of age.

### Objectives

#### Primary objective

To assess the efficacy of a combination corticosteroid/rapid-onset long-acting ß2 agonist (ICS/LABA) budesonide/formoterol inhaler ‘as required’ or, if clinically indicated, ‘both as required, and regularly’ to reduce asthma exacerbations compared with the standard of care for asthma (separate ICS and reliever).

#### Primary endpoint

The primary endpoint will be the number of severe asthma exacerbations in the treatment year, defined as ‘events requiring systemic corticosteroids for three or more days and/or a hospitalisation/emergency room visit for asthma requiring systemic corticosteroids’.

#### Secondary objectives

1. To assess the economic burden and cost-effectiveness of budesonide/formoterol compared with the standard of care (explained below, under title comparator) from the individual, household, health system level.
2. To assess the QoL of children and adolescents on budesonide/formoterol compared with standard of care.

### Trial design

This is a Phase III single-centre, pragmatic open-label randomised controlled trial with two equal sized groups to assess the efficacy, safety, cost effectiveness and QoL of budesonide/formoterol compared with the standard of care over 52 weeks.

Children (6–11 years) and adolescents (12–18 years) with a diagnosis of asthma or symptoms of asthma will be screened for eligibility as per table 1. Those who meet the eligibility criteria will be randomised 1:1 to either receive budesonide/formoterol inhaler or standard of care which includes separate inhalers for symptom relief (short-acting bronchodilator-salbutamol) and maintenance therapy with ICSs (beclomethasone or budesonide) and/or LABA or montelukast as determined by treating physicians.

**Table 1:** Inclusion and exclusion criteria for eligibility for enrolment for AIR-SA 001

Inclusion criteria	Exclusion criteria
Age 6–18 years at the time of consent Known asthmatic on treatment Newly diagnosed asthma based on investigator review and/or medical report All patients will have their asthma diagnosis confirmed (both new or known asthmatic patients) by either spirometry with reversibility or excessive diurnal variability by PEFR twice daily over 2 weeks Ability to perform spirometry and peak expiratory flow rate and/or bronchodilator reversibility testing Only mild or moderate asthma severity, based on medical history At least one exacerbation of asthma in the past year as defined by an event requiring treatment with systemic corticosteroids for ≥3 days and/or a hospitalisation/emergency room visit for asthma requiring treatment with systemic corticosteroids Written consent from the participant or parent/guardian and assent from study participants where applicable Participant and/or parent/guardian agrees to comply with the study procedures, including the completion of the visits and be available for contact telephonically for the non-contact visits	Tuberculosis: active TB disease and contact with people with active TB disease in the last 6 months Chronic sputum expectoration, chest pain, shortness of breath (without any asthma symptoms), dizziness or light-headedness in the last 2 months Cardiac arrhythmia Chronic conditions: thyrotoxicosis, pheochromocytoma, cardiovascular disease, severe hypertension Uncontrolled diabetes mellitus Patients with peak expiratory flow rate <50% of predicted, as these would be classified as severe asthmatics Patients with any history of life-threatening asthma, defined as any history of significant asthma episode(s) requiring intubation associated with hypercapnia, respiratory arrest, hypoxic seizures or asthma-related syncopal episode(s)

PEFR, peak expiratory flow rate.

#### Study definitions

1. Asthma diagnosis will be confirmed by either demonstration of bronchodilator reversibility on spirometry and/or diurnal variability of peak expiratory flow rate over 2 weeks. A bronchodilator response of ≥12% and ≥200 mL will be used to confirm asthma.^19 20^
2. An exacerbation of asthma will be defined as an event requiring treatment with systemic corticosteroids for ≥3 days and/or a hospitalisation/emergency room visit for asthma requiring treatment with systemic corticosteroids, as per American Thoracic Society/ European Respiratory Society (ATS/ERS) definition for exacerbations in clinical trials.^21^
3. Mild asthma is well-controlled with low-intensity treatment, such as as-needed reliever medication alone or with low-dose (ICSs).^16^
4. Moderate asthma is defined as requiring low-dose or medium-dose ICS/LABA for control.^16^

#### Schedule of events

**Figure 1:**
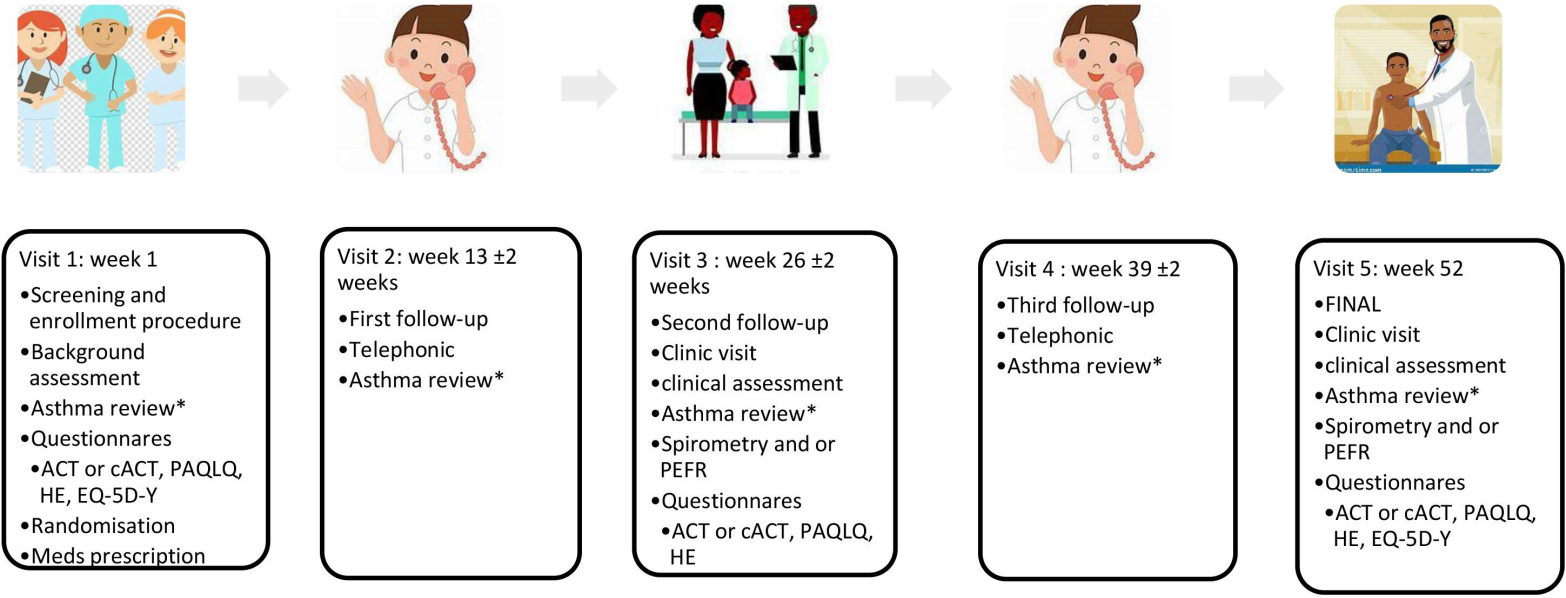
The schedule of events. *Exacerbation, medication changes, additional medication, days lost at work/school, serious adverse events, adverse events. ACT, asthma control test; cACT, childhood control test; EQ-5D-Y, EuroQol Quality of Life questionnaire Youth version; HE, health economics; PAQLQ, Paediatric Asthma Quality of Life Questionnaire; PEFR, peak expiratory flow rate.

All asthma exacerbations and clinic/hospital admissions will be recorded for the duration of the 52-week follow-up. Participants will be given action plan cards on enrolment. After enrolment, week 1, participants will be followed up at 13, 26, 39 and 52 weeks, allowing ±2 weeks window, as well as any unscheduled visits, figure 1. The 13-week and 39-week visits will be telephonic visits to capture the primary outcome and adverse events.

**Figure 2:**
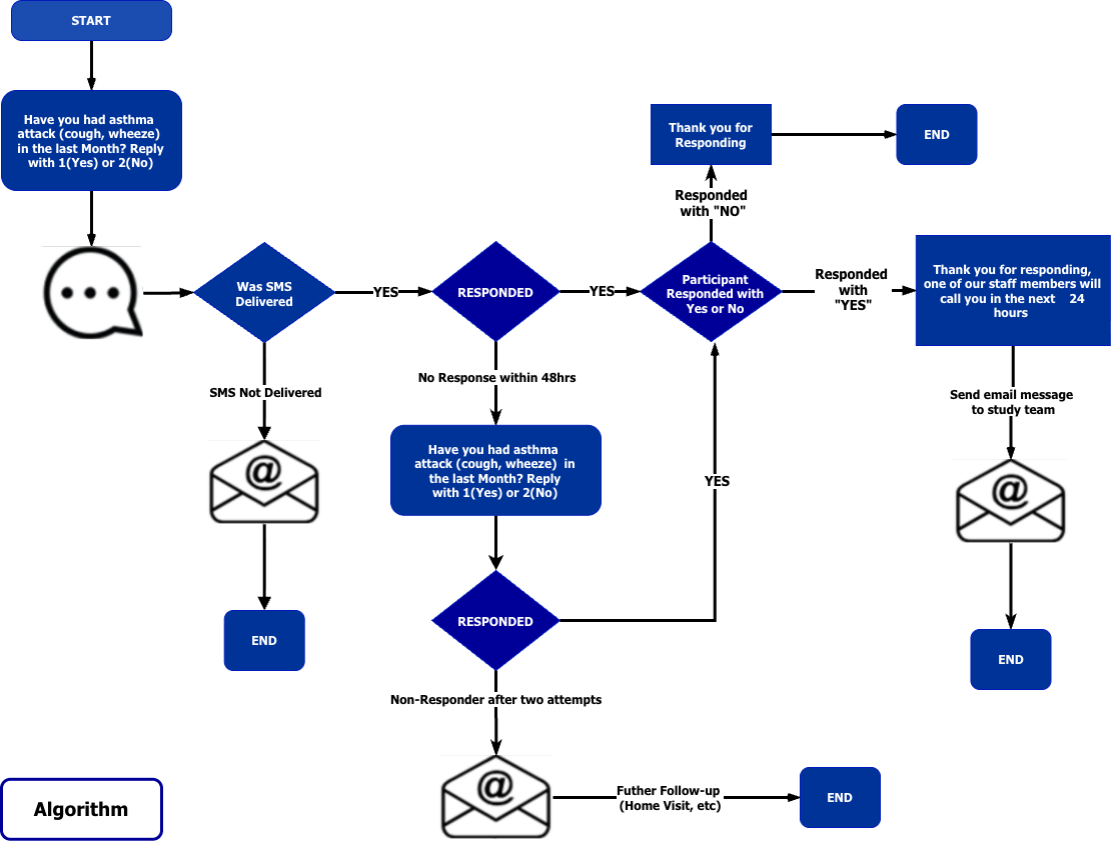
Short message system.

To capture possible asthma exacerbations, participants will be contacted via short message system (SMS) fortnightly or monthly to capture information on the presence or absence of asthma symptoms, hospitalisation or emergency room visits, figure 2. The SMS system will be piloted to see which visit window will be most appropriate. If participants respond as having experienced asthma symptoms, a telephonic follow-up by the study team will be conducted to determine if it is an asthma exacerbation or not and then capture the severity of the exacerbation and record any treatment changes related to the event. If there is no response to two SMSes, the participants will be contacted telephonically to enquire about asthma exacerbations and reason for non-response.

## Methods: participants, interventions and outcomes

### Study setting

Children and adolescents aged 6–18 years with asthma symptoms and/or diagnosis will be recruited from the Africa Health Research Institute (AHRI) Somkhele Research Campus Clinical Trials Unit and participants will be enrolled for six satellite research clinics embedded within primary healthcare facilities, based in a rural community in uMkhanyakude in KwaZulu-Natal.

### Public engagement

The study team plans to have an ongoing involvement of local Community Advisory Board (CAB) and consultation groups. The CAB engagement started during the trial set-up and continued through implementation. The CAB is part of the Trial Steering Committee, involved in 6 monthly engagements. Once the study results are available, these will be presented to CAB, and dissemination plans will be discussed.

### Eligibility criteria

Children and adolescents aged 6–18 years who meet the eligibility criteria. Detailed inclusion and exclusion criteria in table 1.

### Interventions

#### Intervention description

**Figure 3:**
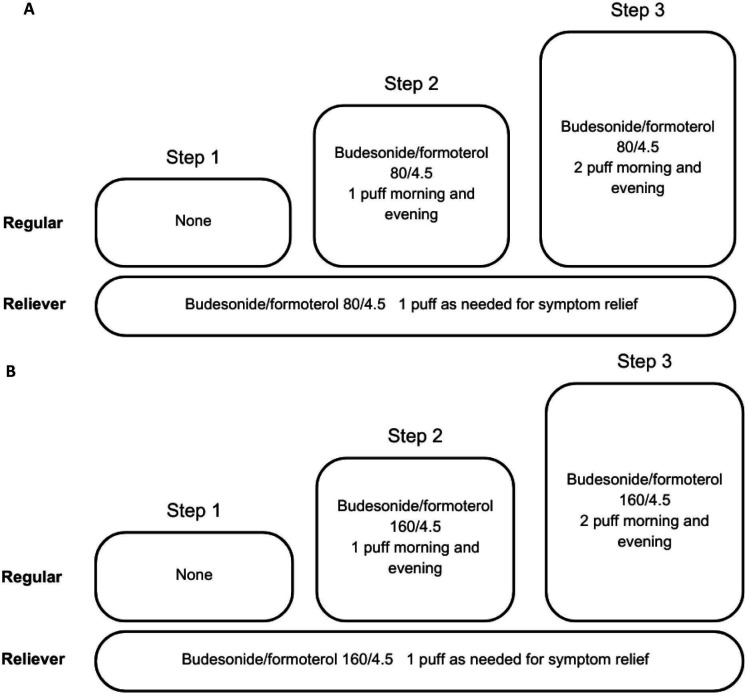
Medication steps for children aged 6–11 years (A) and for adolescents 12–18 years (B)

For the intervention arm, budesonide/formoterol (Symbicort Turbuhaler or Vannair, AstraZeneca) 80/4.5 µg (6–11 years) and 160/4.5 µg (12–18 years) as-needed and /or maintenance. For children 6–11 years of age, the medication will be stepped up and down depending on asthma control every 6 months (figure 3A). For adolescents aged 12–18 years, the medication dosing will be stepped up or down depending on symptom severity and asthma grading every 6 months (figure 3B). The recommended doses are pMDI/DPI 80/4.5 one to two puffs twice daily or one puff as needed (a maximum daily dose of 8 puffs) for children 6–11 years of age and 160/4.5 1–2 inhalations twice daily or one puff as needed (a maximum daily dose of 12 puffs) for adolescents 12–18 years.

#### Comparator

For the control arm, standard of care will be given for children 6–11 years and adolescents 12–18 years. In the current Essential Medicines List in South Africa, the available maintenance anti-inflammatory agents are budesonide or beclomethasone, fluticasone/salmeterol (ICS/LABA) and montelukast (leukotriene receptor antagonist) with salbutamol given as a reliever. The comparator arm will be the standard therapy the patient is receiving and stepping up/down will be as per the treating doctor’s assessment (table 2.

**Table 2:** Standard of care treatment for children and adolescents

Steps	6–11 years	12–18 years Track 1	12–18 years Track 2 (alternative)
1	Low-dose ICS is taken whenever as-needed SABA is taken	Reliever: as needed low dose ICS-formoterol	Low-dose ICS taken whenever as-needed SABA is taken
2	Daily low-dose maintenance ICS plus as-needed SABA	Reliever as needed low dose ICS-formoterol	Low-dose maintenance ICS plus as-needed SABA
3	Low-dose maintenance ICS–LABA and as needed SABA or Medium-dose ICS and as needed SABA or Very low-dose ICS/formoterol MART and as needed SABA	Low-dose maintenance ICS-formoterol. Reliever: as needed low dose ICS-formoterol	Low-dose maintenance ICS–LABA plus as-needed SABA
4	Refer to expert for advice or Medium-dose ICS-LABA and as needed SABA or Low-dose maintenance ICS/formoterol and as-needed SABA	Medium dose maintenance ICS-formoterol. Reliever: as needed low dose ICS-formoterol	Medium/high-dose ICS–LABA plus as-needed SABA
5	Refer for phenotypic assessment ± higher ICS/LABA or add-on-therapy	Add-on LAMA. Refer for phenotypic assessment ± higher ICS/LABA or add-on therapy.	Add-on LAMA. Refer for phenotypic assessment ± higher ICS/LABA or add-on therapy

ICSs, inhaled corticosteroids; LABA, long-acting ß_2_ agonist; LAMA, long-acting muscrinic antagonist; MART, maintenance reliever therapy; SABA, short-acting ß_2_ agonist.

### Adherence training

Adherence counselling is done during enrolment and subsequent follow-up visits. The expiry date is checked by the trial pharmacist, and participants are asked by the pharmacist to return any medication that is close to the expiry date.

### Outcomes

#### Primary outcome

The primary outcome is the number of severe asthma exacerbations per participant per year, defined as ‘events requiring systemic corticosteroids for three or more days and/or a hospitalisation/emergency room visit for asthma requiring systemic corticosteroids’.^21 22^

#### Secondary outcomes

These will include: The Asthma Control Test (>12 years) or childhood Asthma Control Test (6–11 years), Spirometry and the Paediatric Asthma Quality of Life Questionnaires (PAQLQ) to assess QoL will be completed at these scheduled visits 1,3 and 5.

Health economic outcomes will be explored in a subset of 200 participants, ie, 100 in each study arm. The health economics questionnaire will capture data on household income levels, borrowing to fund healthcare, insurance coverage, out-of-pocket healthcare expenditures, caregiver work absences and lost income, loss of household productivity time and education losses due to asthma. Second, these participants will complete the EuroQol Quality of Life questionnaire Youth version (EQ-5D-Y-3L) to capture health-related QoL using validated generic preference-based measures of health. After collecting the health profiles of respondents, we will then use value sets preproduced using standard preference (utility) elicitation. Health system resource use and healthcare costs related to inpatient/out-patient treatment will be collected.

The exploratory outcome is to assess the change in forced expiratory volume in one second prestudy and poststudy. A summary of the outcomes is added as online supplemental material.

### Participants’ timeline

After enrolment, week 1, participants will be followed up at 13, 26, 39 and 52 weeks, allowing ±2 weeks window, as well as any unscheduled visits, see figure 1.

### Sample size

We will enrol 1038 participants. Assuming a 10% loss to follow-up, 1142 participants will complete the trial. The sample size for this trial was designed to detect at least a 25% reduction in the number of exacerbations in the treatment arm relative to the control. Individual variation in the number of exacerbations was accounted for through Poisson regression and the overdispersion parameter k that was set at 0.7. Sample size estimations were conducted in the R programming language (‘power.nb.test’) using the approach of Zhu and Lakkis.^23^ Assuming a two-sided significance level of α=0.05, k=0.7, a 25% reduction in exacerbations in the treatment arm and 90% power, it is envisaged that at least 519 participants would be sufficient to detect the treatment effect.

### Recruitment

Adverts, posters, road shows, department of health clinics, radio interviews and school study presentations will be used to raise awareness about asthma and this clinical trial. Participants will be recruited from the AHRI surrounding clinics in uMkhanyakude district. In an additional recruitment strategy, several primary and secondary schools, in collaboration with the local school health programmes, will screen children for asthma symptoms with an internationally recognised asthma screening questionnaire (Global Asthma Network Questionnaire).^24^ The trial investigators will review participants who are screened positive for asthma, to confirm or exclude an asthma diagnosis in the potential participants and assess their eligibility.

### Methods: assignment of interventions

#### Allocation

Participants will be randomised 1:1 to receive treatment of their asthma. The randomisation schedule will be generated using SAS PROC PLAN, a computer-generated sequence to maintain allocation concealment. Enrolment will be through block randomisation to ensure a balanced representation between the two age groups of children and adolescents. Participants who were newly diagnosed or had previously been initiated on treatment were randomised together and randomly allocated to their treatment arms. A subset of 200 participants, 100 from each arm, will be selected and sequentially numbered, to participate in the health economics questionnaire.

#### Blinding

This trial is an open-labelled study. The principal investigator (designee) who will be assessing the AEs will know the treatment assignment of the participants. Everyone involved in the study is blinded except the statistician.

## Methods: data collection, management and analysis

### Data collection and management

The team will maintain paper or electronic source documentation in a locked place and password encrypted electronic device for all study participants. Protocol-specific participant information will be captured in an electronic care report form (eCRF). The clinical data management system will comply with regulatory guidelines and requirements for electronic systems. Data validation and quality control procedures will be detailed in the data management plan.

All source data and clinical records relating to the study will be archived for a minimum 15-year period after completion of the study in accordance with South African good clinical practice (GCP) guidelines, sponsor and funder requirements. Data will be available for retrospective review or audit by arrangement with the appropriate representative at the archiving organisation (eg, Sponsor Head). A written agreement from the Funder NIHR must precede destruction of the same.

### Statistical methods

#### Statistical analysis

Detailed statistical analysis is added as online supplemental document. For the primary objective, the null hypothesis of no difference in the number of severe asthma exacerbations between the intervention and control arms will be compared using the Poisson regression model with a negative binomial distribution to account for potential overdispersion. Additionally, the null hypothesis of no difference between arms in the median (IQR) number of exacerbations will be determined and compared at each visit using the Kruskal-Wallis test.

For the secondary outcomes, frequencies (proportions) will be determined for categorical asthma control test (ACT) and QoL values and the null hypothesis of no difference will be compared at each visit using the χ^2^ test. Medians (IQRs) will be determined for the continuous scores derived from ACT, QoL and EQ-5D-Y measures as well as the WHO-derived anthropometric and spirometry values. The null hypothesis of no difference between arms at each visit will be compared using the Kruskal-Wallis test. For this analysis, we will conduct our primary analysis ignoring who was a new or existing case. Then we will conduct a sensitivity analysis by controlling for who was a new case and existing case—a binary variable. As this is a real-world study, we understand the limitation. The participants return the medications, so we will have an average dose per participant, except the steroids they received outside the study sites. We will conduct a sensitivity analysis ignoring the dose received, followed by another with an indicator variable indicating who received escalated doses or not—a binary variable.

Health system costs will be used to derive cost per quality-adjusted life year gained or the incremental cost-effectiveness ratio.

#### Descriptive statistics

Means (SD) and medians (IQRs) will be determined for continuous data. Continuous data will also be evaluated for normality by testing the null hypothesis that the data are normally distributed using the Shapiro-Wilk test. Where data are normally distributed, comparisons between the control and treatment arms will be conducted using the two-sample t-test, testing the null hypothesis of no difference. In non-normally distributed data, the Kruskal-Wallis test will be used to test the null hypothesis on no difference between the means of the control and intervention arms. Inferential comparisons will be conducted at each visit.

#### Interim analysis

Interim analysis was done at 50% data collection using O'Brien-Fleming or Pocock boundary.^25^

### Methods: monitoring

#### Composition of the coordinating centr and trial steering committee

The Trial steering committee will provide supervision of the trial and ensure it is delivered in accordance with the South African Guidelines for Good Clinical Practice.^26^ They will meet every 6 months as well as ad hoc if needed.

#### Composition of the data monitoring committee:, its role and reporting structure

The independent Data and Safety Monitoring Board (DSMB) will review the study procedure, ensuring the study participants' safety and the intervention’s efficacy during both the treatment and follow-up periods. The DSMB will evaluate participant safety data throughout the trial, evaluate the efficacy of the study intervention(s) at interim analysis timepoint, and independently provide recommendations to the study sponsor to continue, amend or terminate the trial. AEs will be reported from the time of granting the main study informed consent until the participant’s end of scheduled visit (week 52). AEs occurring after the reporting period that the investigator becomes aware of will be reported to the sponsor within 24 hours of being aware of the event. The DSMB will review all severe adverse events (SAE) on an expedited basis. They will meet once accrual reaches the interim times specified by the O’Brien-Flemming approach or ad hoc, to review study progress.

### Harms

Regardless of severity, all untoward events will be recorded and reported on the eCRF. Each AE will be assessed, graded and managed accordingly. The participants will be followed up until the outcome has been attained. Height in centimetres (cm) and body weight (to the nearest 0.1 kg in light indoor clothing, but without shoes) will be measured, as growth failure assessed by anthropometrics is reported as one of the AEs of systemic steroids.

### Auditing

The audit process will be conducted as frequently as required, independent of the investigators and sponsor. All documents pertinent to this study will be made available for such inspections after providing adequate notice of the intention to audit.

### Criteria for discontinuing

A study participant may withdraw from the study at any time and for any reason without being penalised. The following reasons are the possibilities:

1. At the participant’s request (withdrawal of consent), irrespective of the reason for this.
2. Request from a parent/guardian, irrespective of the reason.
3. At the discretion of the investigator, sponsor, monitor, Biomedical Research Ethics committee (BREC) or South African Health Products Regulatory Authority (SAHPRA) committees.

### Strategies to improve adherence to interventions

Participants will be counselled at enrolment regarding the importance of compliance with therapy. They will also be issued with appointment cards with the dates of the upcoming visits. The site will use an electronic scheduler that will serve for reminding participants who are due and tracking those who have missed a visit. At each follow-up visit (telephonic or clinic), medication adherence will be assessed. After 6 months, participants are asked to return all used and unused inhalers for adherence assessment and counselling.

### Relevant concomitant treatment permitted or prohibited during the trial

Participants will be asked about any concomitant medication at the screening evaluation and at each visit. The drug-to-drug interactions will be assessed. Participants who are enrolled in other clinical trials will be excluded.

### Expected contribution

The expected trial contribution if successful includes health and economics of the patients, households and the institution as well as the recent progress in asthma treatment guidelines.

## Discussion

This trial seeks to assess the efficacy as well as the safety, impact on QoL and cost-effectiveness of budesonide/formoterol in children and adolescents with mild to moderate asthma compared with the standard of care in South Africa. Budesonide/formoterol has transformed asthma treatment in high-income countries for adolescents and adults based on high-quality evidence demonstrating improved symptom control and reduction in the number of exacerbations.^56 9^,^11 27^

In the SYGMA 2 trial in participants with mild asthma, participants in the budesonide–formoterol group had approximately one quarter of the inhaled glucocorticoid exposure of those in the budesonide maintenance group.^6^ Budesonide/formoterol has been shown to reduce exacerbations requiring oral corticosteroids compared with a fixed higher dose of inhaled steroids.^10^

Asthma prevalence in adolescents in South Africa is higher than the global average.^2 31^ Currently, the anti-inflammatory reliever (AIR) and maintenance and reliever therapy (MART) approach is recommended for adults and adolescents with only limited evidence of use for children 6–11 years of age with moderate asthma. There are currently no published studies on the efficacy and safety of the AIR approach in children, and for adolescents, the body of evidence in this age group is much lower than in adults.

While the AIR and MART approach is the current gold standard for adolescents and adults with asthma, the upfront costs of this medication have limited its universal use both in high and low to middle income countries. The upfront cost of medication that limits its universal use is, considering the cost to the purchaser. This affects individuals who are using out-of-pocket funds to get medication and the healthcare system when the medication is stocked in facilities. In South Africa, the health department does provide most medications for free in public facilities to patients (cost incurred by health system), but individuals can also purchase medication at private facilities using out-of-pocket funding or private insurance (cost incurred by individual), so medication costs can be incurred by both.

A cost analysis of its benefit in a LMIC setting with a high disease burden is necessary to inform policy on the cost-effectiveness of this approach for children and adolescents.

### Strength and limitation

The strength of the study is the pragmatic design, inclusion of participants in primary care where most patients with asthma are managed and objective confirmation of asthma by spirometry. As this is a real-world study, we understand there will be limitations. Limitations include lack of blinding, self-reporting of primary outcomes and likelihood of missing dates for self-reported data which increase the risk of social desirability and recall bias. Where missing data are above 5%, multiple imputation techniques assuming an appropriate mechanism of missingness will be used for variables with gaps. The other limitation anticipated is that performance of spirometry may be challenging in younger children. Fractional exhaled nitric oxide will not be measured, which would have assisted in asthma diagnosis and monitoring. The requirement of lung function to confirm diagnosis has the potential to exclude participants who fail to perform spirometry and peak expiratory flow rate. We acknowledge that while we will not have the exact steroid doses, as some are received outside our sites, we will minimise this bias by controlling for whether individuals received the dose or not

### Research ethics approval

The study will be done according to South African GCP Guidelines, the Belmont Report, the Declaration of Helsinki and South Africa legal requirements regarding clinical trials.^32^ The study protocol has been submitted and approved by South African Health Products Regulatory Authority (20231016), University of KwaZulu Natal Biomedical Research Ethics Committee (BREC/00005663/2023), KwaZulu Natal Department of Health KZ_202304_008, South African National Clinical Trial Registry DOH-27-032024-4778, National Human Research Ethics Committee and Clinical Trials, NCT06429475 and Pan African Clinical Trial Research, PACTR202502547023775.

### Protocol amendments

Any protocol deviation or amendments will be submitted to the regulatory and ethics committee. Amendment approval will be sought before any implementation.

### Consent or assent

As per the South African National Department of Health guide on ‘Ethics in Health Research: Principles, Processes and Structures (2015)’, all participants <18 years of age will have to provide assent and consent from the parent or parent/guardian. In South Africa, children ≥18 years of age can independently consent for medical and surgical procedures. Participants aged 18 years of age can independently consent to the clinical trial study. A caregiver in this study is described as a person who factually cares for a child to safeguard the child’s health, well-being and development; and to protect the child from abuse and other harms. Assessment of understanding will be done prior to proceeding with any study procedures. Both assent and consent were translated into IsiZulu. Both will be read to the participants and the parent/caregiver in their preferred language (IsiZulu or English).

### Confidentiality

All electronic devices used during the study conduct will be password-protected, and records will only be accessible to authorised study staff delegated on the delegation log. Confidentiality will be ensured.

### Access to data

The full protocol will be accessible to the relevant stakeholders; however, after completion of the study, the results will be disseminated through conference presentation, publication and in-person presentation. No participants’ identifiers will be shared.

### Provision of post-trial care

There will be no post-trial access to the study drug for any of the participants as this is an investigator-initiated study. Should the findings of the trial be positive for the budesonide–formoterol arm including the cost-effectiveness analysis, these findings will be shared with the Department of Health, particularly the Ministry of Health Chronic Diseases Unit, as part of advocacy efforts to improve access to budesonide–formoterol. The participants will revert to the best standard of care therapy at trial conclusion. Those participants identified to have uncontrolled asthma will be referred to paediatric pulmonologists for further care.

### Dissemination policy

A dissemination plan will be developed with all project partners prior to study completion. After study completion, results will be disseminated using the following strategies: written methods (ie, publications in peer-reviewed scientific journals), presentations at scientific conferences and workshops, in-person dissemination of results to the research participants, the ministry of health, and electronic methods such as the project website and electronic media to publish results.

## Supplementary material

10.1136/bmjresp-2025-003378 online supplemental file 1
