# Supplementary material for: Anti-inflammatory reliever therapy for asthma using inhaled budesonide/formoterol as-needed with or without maintenance in South African children (AIR-SA 001): a description of a randomised clinical trial protocol
Source: BMJ Open Respir Res. 2025 Nov 17;12(1):e003378. doi: 10.1136/bmjresp-2025-003378 (PMC12636877; doi:10.1136/bmjresp-2025-003378)
Supplement: online supplemental file 1 [file bmjresp-12-1-s001.docx]

**Appendices submitted as supplementary documents.**

1. Trial registration
2. Administrative information
3. Consent information
4. Comprehension assessment
5. Abbreviations
6. Summary of the outcome
7. Statistical analysis

**Appendices**

1. Trial registration

This study has been registered and approved by the South African Health Regulatory Authority 20231016, on the 14^th^ Dec 2023, KwaZulu Natal Health Research Committee KZ_202304_008 on the 11^th^ Jan 2024, University of KwaZulu Natal Biomedical Research Ethics Committee BREC/0000/5663/2023 on the 6^th^ Feb 2024, South African Clinical Trials Register DOH-27-032024-4778 on the 14^th^ Mar 2024, ClinicalTrial.gov NCT06429475 on the 20^th^ May 2024 and Pan African Clinical Trial Registry on the 27^th^ Feb 2025, the unique identification number for the registry is PACTR202502547023775 .

1. Administrative information

| Title | Anti-Inflammatory Reliever therapy for asthma using inhaled budesonide/formoterol as-needed with or without maintenance in South African children- AIR-SA 001: A clinical trial protocol. |
| --- | --- |
| Trial registration | This study has been registered and approved by the South African Health Regulatory Authority 20231016, on the 14^th^ Dec 2023, KwaZulu Natal Health Research Committee KZ_202304_008 on the 11^th^ Jan 2024, University of KwaZulu Natal Biomedical Research Ethics Committee BREC/0000/5663/2023 on the 6^th^ Feb 2024, South African Clinical Trials Register DOH-27-032024-4778 on the 14^th^ Mar 2024, ClinicalTrial.gov NCT06429475 on the 20^th^ May 2024 and Pan African Clinical Trial Registry on the 27^th^ Feb 2025, the unique identification number for the registry is PACTR202502547023775 . |
| Protocol Version | 14 Dec 2023, Version 1.5 |
| Funding | This research is funded by the National Institute for Health and Care Research (NIHR), (NIHR302418), awarded grant to Professor Refiloe Masekela, the principal investigator, using United Kingdom (UK) international development funding from the UK Government to support global health research. The views expressed in this publication are those of the author(s) and not necessarily those of the NIHR or the UK government. This financial support is for the overall running of the study including staff, material used and participants re-imbursement. Astra Zeneca has provided support by provision of the investigational products and spacer devices for the duration of the study through a grant. |
| Author details | Hlophe ST^1^, Ndimande N^1^, Mbonigaba J^2^, Bird K^3^, Mkwanazi E^3^, Lebina L^3^, Ngobese N^3^, Otwombe K^4, 5^, Mortimer K*^1,6,7^ and Masekela R*^1,2^  ^1^ *Department of Paediatrics and Child Health, School of Clinical Medicine, Nelson R Mandela School of Medicine, University of KwaZulu-Natal, Durban, South Africa*  ^2^ School of Accounting, Economics and Finance, Department of Economics, *University of KwaZulu-Natal, Durban, South Africa*  *^3^ Africa Health Research Institute, Somkhele Clinical Trials Unit, Mtubatuba, South Africa*  *^4^Perinatal HIV unit, Chris Hani Baragwanath Regional Hospital, Faculty of health sciences, University of Witwatersrand, Johannesburg, South Africa*  *^5^School of Public Health. Faculty of health sciences, University of Witwatersrand, Johannesburg, South Africa*  *^6^Cambridge Africa, Department of Pathology, University of Cambridge, UK*  *^7^Respiratory Medicine, Liverpool University Hospitals NHS Foundation Trust, Liverpool, UK*  All authors contributed to the final review of the manuscript. RM and KM contributed to conceptualization. RM was responsible for funding analysis. EM performed data curation. NN, ST, LL and RM handled project administration. STH, NNN, JM, KB, EM, LL, NN, KM and RM participated in the protocol development. ST, KM and RM were involved in writing original draft. STH, NNN, JM, KB, EM, LL, NN, KO, KM and RM were involved in review and editing. All authors have read, provided feedback, and agreed to the final version of the manuscript for publication.  *STH= ST Hlophe, NNN= N Ndimande, JM= J Mbonigaba, KB= K Bird, EM= E Mkwanazi, LL= L Lebina, KO= K Otwombe, MK=K Mortimer and RM= R Masekela* |
| Name and contact information for the trial sponsor | Dr Pinkie Mekgwe. Chief Operations Officer. Africa Health Research Institute |
| Role of sponsor | The Sponsor (Africa Health Research Institute (AHRI)) has the institutional responsibility to ensure participant safety and undertakes to promptly notify the concerned Investigator/Ethics committee and SAHPRA of findings that could adversely affect the safety of participants included in the study, impact the conduct of the study, alter the BREC’s approval of, or favourable opinion to continue the study. This includes the expedited reporting to these parties of all adverse events that are both serious and unexpected. |
| Principal Investigator | Professor Refiloe Masekela  [masekelar@ukzn.ac.za](mailto:masekelar@ukzn.ac.za) |

1. Informed consent information

**3.1 PARTICIPANT INFORMATION LEAFLET AND INFORMED CONSENT**

Each **participant** must receive, read and understand this document **before** any study-related procedure is performed

| **Study Number:** | AIR-SA-001 |
| --- | --- |
| **Study Title**: | Anti-Inflammatory Reliever therapy for asthma using inhaled budesonide/formoterol as needed with or without maintenance in South African children: A Pragmatic Open Label Phase 3 Randomised Controlled Trial. |
| **Protocol Version and Approval Date:** | Version 1.2; 24 Oct 2023 |
| **Sponsor:** | Africa Health Research Institute. |
| **Investigator (Principal & Site):** | Prof Refiloe Masekela |
| **Institution:** | Africa Health Research Institute |
| **Daytime and After-Hours Telephone Number(s):** | Somkhele +27 (0)35 550 7500 |

**To the potential participant:** *This consent form may contain words that you do not understand. Please ask the study doctor or the study staff to explain any words or information that you do not clearly understand. You may take home* ***an unsigned copy of this consent*** *form to think about or discuss with family or friends* ***before making your decision.***

**ICF administration starting time: _________________ ICF administration finish time: ___________________**

**INTRODUCTION**

Good day, my name is _____________________________________ *(INSERT NAME OF STUDY STAFF)*, I am a ______________________________ *(INSERT DESIGNATION)* at ____________________ (*INSERT SITE).*

I would like to invite you to consider taking part in a research study called “Anti-Inflammatory Reliever therapy for asthma using inhaled budesonide/formoterol as-needed with or without maintenance in South African children: A Pragmatic Open Label Phase 3 Randomised Controlled Trial. “

- Before you decide if you want to be part of this study, we would like to give you information to help you decide if you would like to be part of the study.
- Please take the time to think through the following information and discuss it with others if you wish. Knowing what is involved will help you decide if you want to take part.
- If you have any questions, do not hesitate to ask me.
- You should not agree to take part unless you are happy about all the procedures involved.
- Please be open with me regarding your health history since you may otherwise harm yourself by taking part in this study.
- If you decide to take part in this study, you will be asked to sign this document to confirm that you understand the study. You will also be given a copy to keep and refer to.
- Should you agree to take part in the study we will do so without revealing your study identity.

# What is the purpose of the study?

- The purpose of the study is to check whether using a newer asthma pump will lead to reduced asthma attacks compared to the normally used asthma pump in South African children and adolescents and to see if this will not cost a lot of money.

**Why have I been invited?**

- You have been selected to participate in this study because you have symptoms of asthma and have been diagnosed with asthma. We got your details from Somkhele Campus of the African Health Research Institute (AHRI) database which includes all people living in your area. We are planning to include 1142 participants who will be divided into 2 age groups (6 -11 years) and (12 – 18 years). You will either be in the group that will be taking normal asthma medication dispensed at the clinic (standard medication) or you will receive the study asthma pump (new medicine called Vannair or Symbicort)

# Do I have to take part?

- Taking part in this study is entirely voluntary. You do not have to take part if you would not like to. If you choose to take part and you change your mind later about participation in the study, you can withdraw from the study at any point, without giving a reason. Withdrawal from the study will not affect your clinical care at the clinic or hospital and you will not incur any penalties

# What will happen to me if I decides to take part?

- When you decide to take part, you will be asked to sign the consent form to confirm that you have received enough information about what the study is about and that and you are willing to take part.
- You will be asked a few questions so that we get to know your background better.
- You will be allocated one of the groups then be given an asthma pump (standard one or new one) to use for your asthma management.
- Once allocated, you will stay in that group for the duration of the study.
- The number of participants in the standard asthma pump and the new asthma pump will be the same.
- You will be involved in this study for 12 months. During this period, you will have checkups every 3 months. The check-up will be either by one of the nurses/investigators at the clinic/hospital or will be done telephonically.
- The checkups are to check how you are feeling whether you had any asthma attacks or any undesired harmful effects from the medication or any concerns.
- You can go to the clinic at any point if you are unwell or have any concerns, you don’t have to wait for the scheduled appointments.

# What should I consider?

- You cannot participate in the study if you do not have asthma or if you have active tuberculosis on medication. If you are taking any other medication, you will be expected to let us know what these are. If you are taking the medicine that can interact with any medicine in the new pump, you will not be expected to participate in the study. If you are involved in any other research studies, we will request that you let us know as this may not allow us to enrol you in this current study without discussion with the principal investigator of that study.

# Are there any possible disadvantages or risks to taking part?

- The risks expected during the study are adverse events (side-effects) from the medicines being given, otherwise the project itself is not associated with any risks.

For the new medication that we will be giving for the study.

- The medicine is already recommended for use for the moderate to severe asthma treatment for children 6 to 11 years and for mild asthma treatment in adolescents over the age of 12 years.
- There may be side effects from the drugs. The most common side effects are minor and well-described and include headache, throat irritation, nausea, vomiting, diarrhoea, blocked nose, changes in voice, oral candidiasis (whitish discoloration), nasopharyngitis and upper respiratory tract infections. For the most common side effects, throat irritation and voice changes these can be avoided by rinsing out the mouth after using the medicine.

# What are the possible benefits of taking part?

- All trial participants will benefit from the asthma education and training provided at all health centres, from individualised advice about what action to take in the event of deteriorating asthma and an asthma attack. You will also be guaranteed availability of first-line asthma treatments during the study. If the study findings are positive, we will use the findings to recommend to the health policy makers to make the new medicine available for all children in South Africa.

# Will my family doctor/ General Practitioner be informed of my participation?

- Your family doctor (should you have one) will be provided with the study information sheet and will be informed that you are taking part in the study. We will require him to fill out information for us should you experience an asthma attack and present to him/her for treatment.
- Your family doctor may be contacted if they are any other health concerns that may be picked up during the study.

# Will my taking part in the study be kept confidential?

- Your participation to the study will be known by the relevant people such as clinical team at the clinic you will be attending.
- All trial staff will protect the rights of your information and privacy of your information will be maintained, including the informed consent or assent.
- Arrangements have been made to ensure that information is kept secure, in a locked cupboard accessible on the to the study team.
- Devices with participants details will be encrypted with the password. AHRI and UKZN will maintain all trial records and documents and retain these for at least 15 years.
- For the analysis of data, all direct identifiers to you will be removed, and participants will be identified only by numbers.

# Will I be reimbursed for taking part?

- You will be reimbursed for travel expenses and inconvenience. Your involvement in the study should not cost you any amount.

**What will happen to the data?**

- The information received will be kept in a password crypted devices. Numbers will be used instead of personal identifiers during the data analysis and reporting. The information will be kept by the UKZN and AHRI for 15 years*.*
- We will be using information from AHRI to undertake this study. Research is a task that we perform in the public interest.
- We will use the minimum personally identifiable information.
- We will keep identifiable information about you for 15 years after the study has finished.
- We will store the anonymized research data and any research documents with personal information, such as consent forms, securely at UKZN and AHRI.

*You can find out more about how we use your information by contacting UKZN 031 260 4399/ masekelar@ukzn.ac.za or AHRI* 035 251 0650 *.*

[**What will happen if I don’t want to carry on with the study?**](http://hra-decisiontools.org.uk/consent/content-sheet-support.html#two)

- Participation is voluntary and you may change your minds at a later stage.
- Withdrawal will not affect the care you receive from any health service
- If you withdraw from the study, we will destroy all identifiable samples but will use the data up to your withdrawal.

## **What happens at the end of the study?**

- Your will not be identified from any report or publication placed in the public domain.
- We intend to publish the findings of the study, present it at conferences and give feedback to the community and policy makers.
- Some of the research being undertaken will also contribute to the fulfilment of an educational requirement (e.g. a doctoral thesis).

# What if we find something unexpected?

- If there are unexpected clinical findings, you will be referred to the local clinic/ district hospital. We will need to report these findings, so please inform the study team should these happen.

# What if there is a problem?

- In the event of any problems or concerns/questions you may contact Professor Masekela, at 031 260 4399/ [masekelar@ukzn.ac.za](mailto:masekelar@ukzn.ac.za) or the UKZN Biomedical Research Ethics Committee, contact details as follows:

**BIOMEDICAL RESEARCH ETHICS ADMINISTRATION**

# Research Office, Westville Campus

# Govan Mbeki Building

Private Bag X 54001
Durban
4000

KwaZulu-Natal, SOUTH AFRICA

Tel: 27 31 2602486 - Fax: 27 31 2604609

Email: [BREC@ukzn.ac.za](mailto:ngwenyap@ukzn.ac.za)

- Trial participants and staff will be covered by clinical trial indemnity and insurance for the study.

**How have patients and the public been involved in this study?**

- We plan ongoing involvement of local patient and community advisory board and consultation groups through trial set-up, implementation, and dissemination phases. For example, during the trial set-up phase we sought input into trial plans and wording of participant information and consent forms from the uMkhanyakude District Community Advisory Board and patient representatives. During trial implementation we will share progress reports, discuss, and troubleshoot problems that arise. When the study results are available these will be presented and pathways to impact discussed and planned.

# Who is organizing and funding the study

- The National Institute of Health and Care Research (United Kingdom) via a Global Health Research Professorship Grant has awarded grant to Professor Refiloe Masekela
- Your doctor will not be paid for their role in the study (if contacted to verify information or follow up updates) and there will be no conflicts of interest.

**Who has reviewed the study?**

- We will request ethical review of the trial protocol and other documents by the University of KwaZulu Natal Biomedical Research Ethics Committee and the South African Health Products Regulating Authority (SAHPRA). The trial will not commence until we have ethical approval. The final approved version of the protocol will be registered with Current Controlled Trials Ltd and published in an open access format. Trial oversight committees will be established.

# Further information and contact details:

- Please contact Professor Refiloe Masekela 031 260 4399, [masekelar@ukzn.ac.za](mailto:masekelar@ukzn.ac.za) or in writing to:

4^th^ floor Dept of Paediatrics and Child Health

Nelson R Mandela School of Medicine

Durban

4013

*Thank you for considering taking part.*

**DECLARATION OF CONSENT**

I ……………………………………………………………………………… have been informed about the study entitled ‘Anti-Inflammatory Reliever therapy for asthma using inhaled budesonide/formoterol as-needed with or without maintenance in South African children: A Pragmatic Open Label Phase 3 Randomised Controlled Trial’ by …………………………………………………………………….

I understand the purpose and procedures of the study is to compare different types of medicine in controlling asthma and preventing acute asthma episodes.

I have been given an opportunity to ask questions about the study and have had answers to my satisfaction.

I declare that my participation in this study is entirely voluntary and that I may withdraw at any time without affecting any treatment or care that I would usually be entitled to.

I have been informed about any available compensation or medical treatment if injury occurs to me because of study-related procedures.

If we have any further questions/concerns or queries related to the study, we understand that we may contact the researcher at UKZN/AHRI.

If I have any questions or concerns about my rights as a study participant, or if I am concerned about an aspect of the study or the researchers then I may contact:

**BIOMEDICAL RESEARCH ETHICS ADMINISTRATION**

# Research Office, Westville Campus

# Govan Mbeki Building

Private Bag X 54001
Durban
4000

KwaZulu-Natal, SOUTH AFRICA

Tel: 27 31 2602486 - Fax: 27 31 2604609

Email: [BREC@ukzn.ac.za](mailto:ngwenyap@ukzn.ac.za)

**SOUTH AFRICAN HEALTH PRODUCTS REGULATORY AUTHORITY:**

The Chief Executive Officer

South African Health Products Regulatory Authority

Loftus Park

Building A

402 Kirkness Street

Arcadia, Pretoria

0083

E-mail: [Boitumelo.Semete@sahpra.org.za](mailto:Boitumelo.Semete@sahpra.org.za)

Tel: 012 501 0413

**PARTICIPANT**:

__________________________________________________________________________________________

Printed Name(s) and Surname

__________________________________________________________________________________________

Signature / Mark or Thumbprint Date and Time

**INFORM CONSENT ADMINISTRATOR:**

__________________________________________________________________________________________

Printed Name(s) and Surname

__________________________________________________________________________________________

Signature / Mark or Thumbprint Date and Time

**WITNESS** (If applicable)**:**

__________________________________________________________________________________________

Printed Name(s) and Surname

__________________________________________________________________________________________

Signature / Mark or Thumbprint Date and Time

**INVESTIGATOR:**

__________________________________________________________________________________________

Printed Name(s) and Surname

__________________________________________________________________________________________

Signature Date and Time

**3.2 PARTICIPANT INFORMATION LEAFLET AND ASSENT FORM**

Each **participant** must receive, be informed and/or read and understand this document **before** any study-related procedure is performed

| **Study Number:** | AIR-SA-001 |
| --- | --- |
| **Study Title**: | Anti-Inflammatory Reliever therapy for asthma using inhaled budesonide/formoterol as-needed with or without maintenance in South African children: A Pragmatic Open Label Phase 3 Randomised Controlled Trial. |
| **Protocol Version and Approval Date:** | Version 1.2; 24 October 2023 |
| **Sponsor:** | Africa Health Research Institute. |
| **Investigator (Principal & Site):** | Prof Refiloe Masekela |
| **Institution:** | Africa Health Research Institute |
| **Daytime and After-Hours Telephone Number(s):** | Daytime:+27 (0)35 550 7500 After-Hours 079 056 4232 |

**To the potential participant:** *This consent form may contain words that you do not understand. Please ask the study doctor or the study staff to explain any words or information that you do not clearly understand. You may take home* ***an unsigned copy of this consent*** *form to think about or discuss with family or friends* ***before making your decision.***

**ICF administration starting time: _________________ ICF administration finish time: ___________________**

**INTRODUCTION**

Good day, my name is _____________________________________ *(INSERT NAME OF STUDY STAFF)*, I am a ______________________________ *(INSERT DESIGNATION)* at ____________________ (*INSERT SITE).*

I would like to invite you to consider taking part in a research study called “Anti-Inflammatory Reliever therapy for asthma using inhaled budesonide/formoterol as-needed with or without maintenance in South African children: A Pragmatic Open Label Phase 3 Randomised Controlled Trial. “

- Before you decide if you want to be part of this study, we would like to give you information to help you decide if you would like to be part of the study.
- Please take the time to think through the following information and discuss it with others if you wish. Knowing what is involved will help you decide if you want to take part.
- If you have any questions, do not hesitate to ask me.
- You should not agree to take part unless you are happy about all the procedures involved.
- Please be open with me regarding your health history since you may otherwise harm yourself by taking part in this study.
- If you decide to take part in this study, you will be asked to sign this document to confirm that you understand the study. You will also be given a copy to keep and refer to.
- Should you agree to take part in the study we will do so without telling anyone who you are or your study identity.

**What is the study about?**

- The reason for the study is about my asthma and to check whether using a newer asthma pump will may cause fewer asthma attacks compared to the normally used asthma pump and will cost less. You will either get the new pump or use the normal (regular) asthma medication from the clinic.

**Why have I been invited?**

- You have been invited because you have asthma. We are going to compare those who will be on medication the normally used at your clinic or hospital (standard) asthma pump and new asthma pump (Vannair or Symbicort).

**Do I have to take part?**

- No, you do not have to take part. If you agree to take part you are also free to quit the study at any time and this will not affect your care at the clinic or hospital.

**What will happen to me if I decide to take part?**

- When you decide to take part, you will be given the asthma medicine that you have been allocated to. You will take the medicine for a year in the group you are allocated to. You will have check-ups at the clinic twice and you will have two telephone visit with the study team.
- The nurses will ask you questions about your asthma, your medicines you used and any attacks you may have had.

**Will I have bad effects from the medicine?**

- The new medicine for asthma can make you feel slightly ill. You can feel unwell, want to vomit or have a blocked nose, sore head or change in your voice. If you are unwell let the study team know you are not well. Should you feel very bad from the medicine the doctor will check you and decide if the medicine should be stopped or not.
- The medicine can also make whitish discolouration inside your mouth. You must rinse your mouth after taking this medicine to reduce the risk.

**How will the study help?**

- If we see that the new medicine works better that the normal medicine in the clinic, we will recommend that the new medicine should be given to children with asthma in South Africa.

**Will my taking part in the study be kept confidential?**

- No one but the study team will know what answers you have given and all the your name and information will not be available to anyone but the study team.

**What if there is a problem or questions?**

- In the event of any problems or concerns/questions you can speak to Professor Masekela, at 0312604399/ [masekelar@ukzn.ac.za](mailto:masekelar@ukzn.ac.za) or the UKZN Biomedical Research Ethics Committee, or South African Health Products Regulatory Authority contact details as follows:

**BIOMEDICAL RESEARCH ETHICS ADMINISTRATION**

Research Office, Westville Campus

Govan Mbeki Building

Private Bag X 54001  
Durban  
4000

KwaZulu-Natal, SOUTH AFRICA

Tel: 27 31 2602486 - Fax: 27 31 2604609

Email: [BREC@ukzn.ac.za](mailto:ngwenyap@ukzn.ac.za)

**SOUTH AFRICAN HEALTH PRODUCTS REGULATORY AUTHORITY:**

The Chief Executive Officer

South African Health Products Regulatory Authority

Loftus Park

Building A

402 Kirkness Street

Arcadia, Pretoria

0083

E-mail: [Boitumelo.Semete@sahpra.org.za](mailto:Boitumelo.Semete@sahpra.org.za)

Tel: 012 501 0413

**PARTICIPANT ASSENT**:

__________________________________________________________________________________________

 Printed Name(s) and Surname

__________________________________________________________________________________________

Signature / Mark or Thumbprint Date and Time

**INFORM CONSENT ADMINISTRATOR:**

__________________________________________________________________________________________

 Printed Name(s) and Surname

__________________________________________________________________________________________

Signature / Mark or Thumbprint                               Date and Time

**WITNESS** (If applicable)**:**

__________________________________________________________________________________________

 Printed Name(s) and Surname

__________________________________________________________________________________________

Signature / Mark or Thumbprint Date and Time

**INVESTIGATOR:**

__________________________________________________________________________________________

 Printed Name(s) and Surname

__________________________________________________________________________________________

Signature Date and Time

1. Comprehension assessment

**AIR SA-001 Informed Assent Comprehension Assessment**

| **NAME** |  | **DATE** |  |
| --- | --- | --- | --- |

| **Instructions** |  | **Comment Code** | |
| --- | --- | --- | --- |
| The assessment should be administered by the study staff member to the potential participant after the informed consent discussion is completed but before the participant is asked to sign or mark the informed consent form. The staff member administering the assessment should read the questions/statements below and mark the required points of comprehension. |  | **A** | Answered correctly on first try |
|  |  | **B** | Could not answer at first but answered correctly with probing |
|  |  | **C** | Answered incorrectly at first but answered correctly after discussion |
|  |  | **D** | Not able to answer correctly currently |
|  |  | **E** | Other (describe) ____________________________________ |

| **Open-Ended Question/Statement** | | **Required Points of Comprehension** | **Assessed (✓)** | **Comments**  **(Enter code or other notes)** |
| --- | --- | --- | --- | --- |
| **1** | **Please tell me your understanding of the purpose of the study.** | The study is about my asthma and to check whether using a newer asthma pump may cause fewer asthma attacks compared to the normally used asthma pump and will cost less |  |  |
| **2** | **How will you take the medicine during the study and how often will you take them** | I will use the pump once or twice a day or when needed. |  |  |
| **3** | **How long will I participate in the study for?** | 52 weeks (12 months) |  |  |
| **4** | **Besides taking the medications, what else are participants being asked to do in this study?** | Come to the clinic for regular visits |  |  |
|  |  | My caregiver and I to record asthma attacks and report when asked telephonically every month |  |  |
|  |  | My caregiver and I to complete questionnaires about my asthma symptoms |  |  |
| **5** | **What are the possible risks of being a participant in the study?** | The new pump can make me feel slightly jittery It can also make whitish discolouration inside my mouth, so I need to rinse my mouth after using the spray/powder |  |  |
| **6** | **What are possible benefits for participants in the study?** | If the new medicine works better that the normal medicine in the clinic, the study doctor will recommend that it becomes available for children |  |  |
| **7** | **What happens if you become pregnant during the study?** | The study doctor will discuss possible risks and benefits of staying in the study |  |  |
|  |  | I can choose to stay in the study if I want to |  |  |
| **8** | **What should participants do if they have questions or concerns about the study or their health?** | I how to contact study staff, ethics team (i.e. by phone, by email or return to clinic) |  |  |
| **9** | **What will happen if a participant decides not to join the study or stop the study?** | I can choose to join the study or not. The decision will have no change to their health care. |  |  |
|  |  | I can choose to drop out of the study at any point with no change to their health care. |  |  |
| **10** | **How will information about participants in the study be protected?** | Information about me will be kept confidential |  |  |
|  |  | Only people working on the study have access to her information |  |  |

| **Outcome** |
| --- |
| - Demonstrated comprehension of all required points, decided to enrol in study. - Demonstrated comprehension of all required points, decided NOT to enrol in study. - Demonstrated comprehension of all required points, deferred enrolment decision. - Did not demonstrate comprehension of all required points (yet), needs more time/discussion. - Unable to demonstrate comprehension of all required points, consent process discontinued. - Other (specify)___________________________________________________________ |
|  |
|  |
|  |
|  |

| **Staff Signature** |  | **Staff Date** |  |
| --- | --- | --- | --- |

**AIR-SA-001 Informed consent Comprehension Assessment**

| **NAME** |  | **DATE** |  |
| --- | --- | --- | --- |

| **Instructions** |  | **Comment Code** | |
| --- | --- | --- | --- |
| The assessment should be administered by the study staff member to the potential participant after the informed consent discussion is completed but before the participant is asked to sign or mark the informed consent form. The staff member administering the assessment should read the questions/statements below and mark the required points of comprehension. |  | **A** | Answered correctly on first try |
|  |  | **B** | Could not answer at first but answered correctly with probing |
|  |  | **C** | Answered incorrectly at first but answered correctly after discussion |
|  |  | **D** | Not able to answer correctly currently |
|  |  | **E** | Other (describe) ____________________________________ |

| **Open-Ended Question/Statement** | | **Required Points of Comprehension** | **Assessed (ü)** | **Comments**  **(Enter code or other notes)** |
| --- | --- | --- | --- | --- |
| **1** | **Please tell me your understanding of the purpose of the study.** | To determine if the newer asthma pump will reduce asthma attacks compared to the usual asthma pump and to determine if this will not cost a lot of money |  |  |
| **2** | **How will you take the medicine during the study and how often will you take them** | An asthma pump once or twice a day and/or when needed |  |  |
| **3** | **How long will I participate in the study for?** | 52 weeks (12 months) |  |  |
| **4** | **Besides taking the medications, what else are participants being asked to do in this study?** | Come to the clinic for regular visits (2 times after enrolment) |  |  |
|  |  | Have telephonic consultation 2 scheduled and messages once to twice a month followed by telephonic call if needed |  |  |
|  |  | Complete questionnaires about participants asthma symptoms, quality of life and health economics |  |  |
| **5** | **What are the possible risks of being a participant in the study?** | The most common side effects include feeling a little jittery at the beginning and this will get better and whitish discoloration in the throat, my child must rinse his/her mouth after using the asthma pump. |  |  |
| **6** | **What are possible benefits for participants in the study?** | All trial participants will benefit from the asthma education and training provided at all health centres, from individualised advice about what action to take in the event of deteriorating asthma and an asthma attack.  New pump will be available during the study.  If the study findings are positive, study doctor will use the findings to recommend to the health policy makers to make the new medicine available for all children in South Africa. |  |  |
| **7** | **What happens if you become pregnant during the study?** | Participants study doctor will discuss possible risks and benefits of staying in the study |  |  |
|  |  | Participants can choose to stay in the study if they want to |  |  |
| **8** | **What should participants do if they have questions or concerns about the study or their health?** | Participant must state how to contact study staff and ethics team (i.e. by phone, by email, return to clinic) |  |  |
| **9** | **What will happen if a participant decides not to join the study or stop the study?** | Participants can choose to join the study or not. The decision will have no change to their health care. |  |  |
|  |  | Participants can choose to drop out of the study at any point with no change to their health care. |  |  |
| **10** | **How will information about participants in the study be protected?** | Information about participants will be kept confidential |  |  |
|  |  | Only people working on the study have access to her information |  |  |

| **Outcome** |
| --- |
| - Demonstrated comprehension of all required points, decided to enrol in study. - Demonstrated comprehension of all required points, decided NOT to enrol in study. - Demonstrated comprehension of all required points, deferred enrolment decision. - Did not demonstrate comprehension of all required points (yet), needs more time/discussion. - Unable to demonstrate comprehension of all required points, consent process discontinued. - Other (specify)___________________________________________________________ |
|  |
|  |
|  |
|  |

| **Staff Signature** |  | **Staff Date** |  |
| --- | --- | --- | --- |

1. Abbreviations

| **Abbreviation** | **Definition** |
| --- | --- |
| ACT | Asthma Control Test |
| AE | Adverse Event |
| AIR | Anti-Inflammatory Reliever |
| AIR-SA 001 | Anti-Inflammatory Reliever therapy for asthma using inhaled budesonide/formoterol as-needed with or without maintenance in South African children |
| AHRI | Africa Health Research Institute |
| ATS | American Thoracic Society |
| BDR | Bronchodilator responsiveness |
| BREC | Biomedical Research Ethics Committee |
| cACT | Childhood Asthma Control Test |
| CDMS | Clinical Data Management System |
| CTU | Clinical Trials Unit |
| DMP | Data Management Plan |
| DOH | Department of Health |
| DSMB | Data Safety Monitoring Board |
| ERS | European Respiratory Society |
| EQ-5D-Y | Euro Quality of Life Youth version |
| FEV1 | Forced Expiratory Volume in one second |
| GAN | Global Asthma Network |
| GCP | Good Clinical Practice |
| GINA | Global Initiative for Asthma |
| GPP | Good Pharmacy Practice |
| HE | Health Economics |
| HIC | High Income Countries |
| ICS | Inhaled Corticosteroids |
| IMP | Investigational Medicinal Product |
| LABA | Long-Acting Beta -2- Agonist |
| LMIC | Low to Middle Income Countries |
| MART | Maintenance and Reliever Therapy |
| NCD | Non-Communicable Disease |
| PAQLQ | Paediatric Asthma Quality of Life Questionnaire |
| PEFR | Peak Expiratory Flow Rate |
| QOL | Quality of Life |
| REDCap | Research Electronic Data Capture |
| SABA | Short Acting Beta Agonist |
| SAE | Serious Adverse Events |
| SA GCP | South African Good Clinical Practice |
| SAHPRA | South African Health Products Regulatory Authority |
| SMART | Symbicort Maintenance and Reliever Therapy |
| SMS | Short Message System |
| sSA | Sub-Saharan Africa |
| SOE | Schedule of Events |
| SOP | Standard Operating Procedures |
| TB | Tuberculosis |
| TSC | Trial Steering Committee |
| UK | United Kingdom |
| UKZN | University of KwaZulu-Natal |
| WHO | World Health Organization |

1. **Table 3: Summary of the study outcomes**

| Primary outcome | Number of severe asthma exacerbations per year per individual |
| --- | --- |
| Secondary outcomes | Asthma control test/childhood asthma control test |
|  | Paediatric Asthma Control Quality of Life Questionnaires |
|  | Health economics |
|  | EuroQol Quality of Life Questionnaire Youth version |
|  | Days lost at school or work |

1. **Statistical methods**

*Statistical analysis*

For the primary objective, the null hypothesis of no difference in the number of severe asthma exacerbations between the intervention and control arms will be compared using the Poisson regression model with a negative binomial distribution to account for potential overdispersion. Additionally, the null hypothesis of no difference between arms in the median (interquartile range) number of exacerbations will be determined and compared at each visit using the Kruskal-Walli’s test.

For the secondary outcomes, frequencies (proportions) will be determined for categorical ACT and QoL values and the null hypothesis of no difference will be compared at each visit using the Chi-square test. Medians (interquartile ranges) will be determined for the continuous scores derived from ACT, QoL and EQ-5D-Y measures as well as the WHO derived anthropometric and spirometry values. The null hypothesis of no difference between arms at each visit will be compared using the Kruskal-Walli’s test.

Repeated measures over time will be compared by treatment arms for both continuous and categorical dependent variables. Linear mixed-effects modelling will be utilized for continuous dependent data whereas generalised estimating equation will evaluate categorical variables. Continuous repeated measures data will be analysed using the linear mixed effects model incorporating the treatment arm as a covariate. Categorical measures collected over time will be analysed using the generalised estimating equation.

Pre and post-test analysis testing the null hypothesis that the mean/median difference in FEV1 measures within arms is zero will be analysed using the paired sample t-test or Wilcoxon signed-rank test as appropriate. For safety adverse events, incidence (95% confidence intervals) per 100-person years will be determined and compared using the Poisson regression model. The Kaplan-Meier curve will be fitted to test the null hypothesis of no difference in the time to first AE and SAE.

Health system costs will be used to derive cost per quality-adjusted life year (QALY) gained or the incremental cost-effectiveness ratio (ICER).

Statistical analysis will be conducted using the software SAS, R and Stata.

*Descriptive Statistics*

Means (standard deviations) and medians (interquartile ranges) will be determined for continuous data. Continuous data will also be evaluated for normality by testing the null hypothesis that the data is normally distributed using the Shapiro-Wilks test. Where data are normally distributed, comparisons between the control and treatment arms will be conducted using the two-sample t-test, testing the null hypothesis of no difference. In non-normally distributed data, the Kruskal-Wallis test will be used to test the null hypothesis on no difference between the means of the control and intervention arms. Inferential comparisons will be conducted at each visit. For categorical variables, frequencies will be determined and reported together with their percentages/proportions. The Chi-square test will be used to test the null hypothesis of no association between the control and treatment arms at each visit. Continuous data collected over time will be analysed, to compare treatment and control arms using the linear mixed modelling under the generalised linear model framework. For categorical data collected over time, comparison between treatment arms will be done using the generalised estimating equation. Both longitudinal analysis approaches consider within and between person differences through the covariance structures.

*Comparative Analysis*

Primary Outcome: For the primary outcome testing the null hypothesis of no difference in the number of exacerbations between arms, the Poisson regression model with a negative binomial distribution will be used to compare the groups with treatment status as the primary predictor, a log-link function and an offset variable derived from person time. The negative binomial distribution plays a useful role in the presence of overdispersion. This approach will estimate rate ratios (and their 95% confidence intervals) for exacerbation holding the control group as a reference. Model fit will be evaluated using Deviance and Pearson Chi-square statistics.

*Interim Analysis*

At 50% data collection using O'Brien-Fleming or Pocock boundary.
